# The role of mechanotransduction versus hypoxia during simulated orthodontic compressive strain—an in vitro study of human periodontal ligament fibroblasts

**DOI:** 10.1038/s41368-019-0066-x

**Published:** 2019-11-05

**Authors:** Niklas Ullrich, Agnes Schröder, Jonathan Jantsch, Gerrit Spanier, Peter Proff, Christian Kirschneck

**Affiliations:** 1 University Medical Centre of Regensburg, Franz-Josef-Strauß-Allee 11, D-93053 Regensburg, Germany; 2 Department of Orthodontics, University Medical Centre of Regensburg, Franz-Josef-Strauß-Allee 11, D-93053 Regensburg, Germany; 3 Department of Medical Microbiology and Hygiene, University Medical Centre of Regensburg, Franz-Josef-Strauß-Allee 11, D-93053 Regensburg, Germany; 4 Department of Cranio-Maxillo-Facial Surgery, University Medical Centre of Regensburg, Franz-Josef-Strauß-Allee 11, D-93053 Regensburg, Germany

**Keywords:** Molecular medicine, Orthodontics

## Abstract

During orthodontic tooth movement (OTM) mechanical forces trigger pseudo-inflammatory, osteoclastogenic and remodelling processes in the periodontal ligament (PDL) that are mediated by PDL fibroblasts via the expression of various signalling molecules. Thus far, it is unknown whether these processes are mainly induced by mechanical cellular deformation (mechanotransduction) or by concomitant hypoxic conditions via the compression of periodontal blood vessels. Human primary PDL fibroblasts were randomly seeded in conventional six-well cell culture plates with O_2_-impermeable polystyrene membranes and in special plates with gas-permeable membranes (Lumox®, Sarstedt), enabling the experimental separation of mechanotransducive and hypoxic effects that occur concomitantly during OTM. To simulate physiological orthodontic compressive forces, PDL fibroblasts were stimulated mechanically at 2 g·cm^−2^ for 48 h after 24 h of pre-incubation. We quantified the cell viability by MTT assay, gene expression by quantitative real-time polymerase chain reaction (RT-qPCR) and protein expression by western blot/enzyme-linked immunosorbent assays (ELISA). In addition, PDL-fibroblast-mediated osteoclastogenesis (TRAP^+^ cells) was measured in a 72-h coculture with RAW264.7 cells. The expression of HIF-1α, COX-2, PGE2, VEGF, COL1A2, collagen and ALPL, and the RANKL/OPG ratios at the mRNA/protein levels during PDL-fibroblast-mediated osteoclastogenesis were significantly elevated by mechanical loading irrespective of the oxygen supply, whereas hypoxic conditions had no significant additional effects. The cellular–molecular mediation of OTM by PDL fibroblasts via the expression of various signalling molecules is expected to be predominantly controlled by the application of force (mechanotransduction), whereas hypoxic effects seem to play only a minor role. In the context of OTM, the hypoxic marker HIF-1α does not appear to be primarily stabilized by a reduced O_2_ supply but is rather stabilised mechanically.

## Introduction

In the dental specialty of orthodontics, removable or fixed orthodontic appliances are used for the treatment of malocclusions to move malpositioned teeth to the correct position. Mechanical orthodontic forces create compression and tension areas in different regions of the periodontal ligament.^1^ Whereas tension areas are characterized by increased bone formation, bone resorption processes take place in pressure areas.^1^

Human periodontal ligament (hPDL) fibroblasts are the predominant cells within the periodontal ligament.^2^ They are responsible for the regulation of tissue homoeostasis and the formation of collagenous structural proteins, and play a regulatory role in innate immune defence.^1,2^ These cells also play an important mediating role during orthodontic tooth movement (OTM)^1,2^ and have thus been intensively investigated in basic orthodontic research,^3–7^ especially with regard to their responses to compressive or tensile orthodontic forces or periodontal pathogens and their toxins.^8–10^

In compression areas of the periodontal ligament during orthodontic force application, hPDL fibroblasts become mechanically deformed (mechanotransduction), and thus mechanosensitive receptors and ion channels in the cell membrane are predicted to be stimulated.^11^ A hPDL-mediated sterile pseudo-inflammatory reaction induces increased expression of IL-6, IL-8 and COX-2, followed by enhanced extracellular matrix remodelling and bone resorption triggered by increased receptor activator of NF-κB ligand (RANKL) and reduced osteoprotegerin (OPG) expression.^1,7^ On the other hand, the concomitant compression of blood vessels during OTM disturbs circulation in compressive areas of the periodontal ligament,^12^ reducing the local oxygen supply within the PDL (hypoxia).^13^ It has been postulated that this local reduction of the O_2_ supply may play a significant role in the cellular regulation of orthodontic tooth movement.^13^

Although the molecular and cellular processes enabling OTM that are mediated by hPDL fibroblasts under mechanical load have been studied before,^3,7,14^ it is still unclear whether these processes are mainly triggered by the mechanical deformation of hPDL fibroblasts (mechanotransduction) during orthodontic force application or by concomitant hypoxia resulting from the compression of periodontal blood vessels and a disturbance in the local blood flow. Understanding the impact of mechanotransduction vs. that of hypoxia on the molecular processes enabling OTM could, in the long term, lead to the development of new therapeutic and preventive options for orthodontic treatment.

In this study, we thus investigated the expression patterns of genes and proteins that were previously identified to be associated with OTM^1,15,16^ after compressive force application in the presence of a normal or reduced oxygen supply by using conventional six-well cell culture plates or special cell culture dishes with gas-permeable membranes to gain a better understanding of the respective roles of mechanotransduction and oxygen supply in the molecular and cellular processes occurring in compressive areas of the periodontal ligament during OTM.

## Results

### Effects of mechanotransduction vs. hypoxia on hPDL cell number and viability

**Fig. 1:**
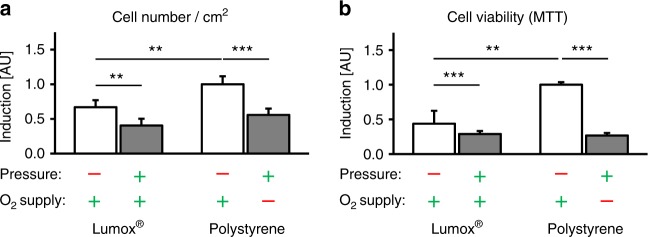
Cell number per cm^2^ (**a**) and cell viability of hPDL fibroblasts (**b**, MTT assays) after 72 h of incubation. *N* = 2, *n* = 6. Bars indicate mean values ± standard deviation. **P ≤ 0.01, ***P ≤ 0.001

Mechanotransduction (pressure) caused a significant reduction both in cell number per cm^2^ (*P* ≤ 0.007, Fig. 1a) and in cell viability (*P* ≤ 0.001, Fig. 1b), which was more pronounced in the presence of reduced O_2_ supply, particularly regarding cell viability.

### Effects of mechanotransduction vs. hypoxia on the hPDL expression pattern and HIF-1α stabilization

The Lumox® and polystyrene control groups (under normoxia and no pressure) did not show significant expression differences for any of the evaluated genes/proteins (*P* ≥ 0.086) or for HIF-1α stabilization (*P* = 0.081).

**Fig. 2:**
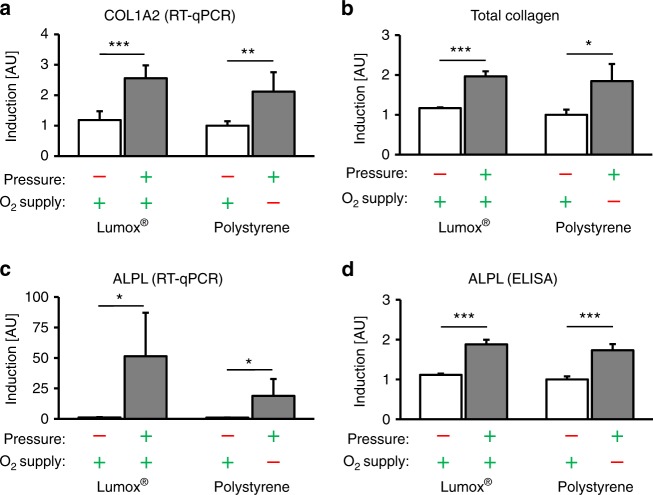
Effects of mechanotransduction vs. oxygen supply on the hPDL fibroblast expression pattern. **a** COL1A2 mRNA, **b** total collagen, **c** ALPL mRNA and **d** ALPL protein expression in the presence of pressure under normoxic (Lumox®) and hypoxic (polystyrene) conditions (*N* = 3, *n* = 9). Bars indicate mean values ± standard deviation. *P ≤ 0.05, **P ≤ 0.01, ***P ≤ 0.001. AU = arbitrary units

The expression of COL1A2 (collagen 1 alpha-2) was significantly enhanced by compressive force application, independent of the O_2_ supply (*P* ≤ 0.003, Fig. 2a). The level of O_2_ supply during compression, however, had no significant effect on COL1A2 gene expression (*P* = 0.348). Increased levels of COL1A2 gene expression also resulted in an increased quantity of total collagen with (*P* = 0.019) or without O_2_ restriction (*P* < 0.001, Fig. 2b). Altering the O_2_ supply during pressure application did not affect this observation (*P* = 0.906).

Alkaline phosphatase (ALPL) gene expression was also upregulated by compressive forces during normoxia (*P* = 0.013) and hypoxia (*P* = 0.02, Fig. 2c). In addition, a reduced O_2_ supply during cell compression seemed to attenuate pressure-induced ALPL upregulation (*P* = 0.110) at the transcriptional level. ALPL at the protein level showed O_2_-independent accumulation after mechanical compression (*P* < 0.001, Fig. 2d). The apparent attenuation of pressure-induced ALPL expression by a reduced O_2_ supply was not reflected at the protein level, and a reduction in the O_2_ supply did not alter the ALPL protein accumulation (*P* = 0.384).

**Fig. 3:**
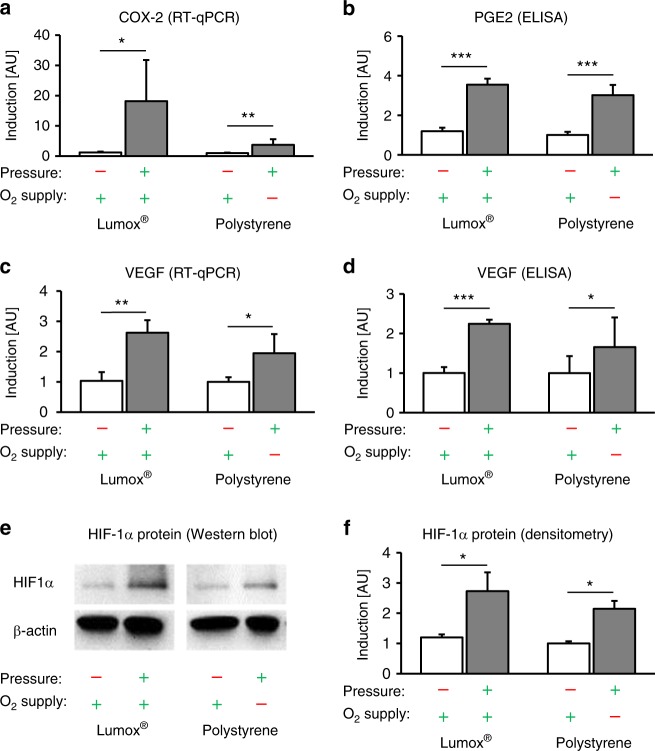
Effects of mechanotransduction vs. oxygen supply on hPDL fibroblast expression patterns and HIF-1α stabilization. **a** COX-2 mRNA, **b** PGE2 protein, **c** VEGF mRNA and **d** VEGF protein expression in the presence of pressure under normoxic (Lumox®) and hypoxic (polystyrene) conditions (*N* = 3, *n* = 9). **e** Representative immunoblot of HIF-1α protein expression. **f** Densitometric immunoblot analysis of HIF-1α protein expression (*N* = 4). Bars indicate mean values ± standard deviation. *P ≤ 0.05, **P ≤ 0.01, ***P ≤ 0.001. AU = arbitrary units

The expression of the proinflammatory gene cyclooxygenase 2 (COX-2) was significantly upregulated by compressive force application (mechanotransduction) independent of the O_2_ supply (*P* ≤ 0.024, Fig. 3a). However, the upregulation of COX-2 by pressure in combination with a reduced O_2_ supply was less pronounced compared with the change in the relative COX-2 gene expression during pressure application during normoxia (*P* = 0.052). This mechanically induced COX-2 gene expression was also associated with the significantly increased protein expression of PGE2 after compressive force application regardless of the level of O_2_ supply (*P* < 0.001, Fig. 3b). Reducing the O_2_ supply during compression had no effect on PGE2 expression (*P* = 0.223).

Compressive force application increased the expression of vascular endothelial growth factor (VEGF) significantly, independently of the O_2_ supply (*P* ≤ 0.015, Fig. 3c). A reduced O_2_ supply during compression, by contrast, had no additional significant effect on VEGF gene expression (*P* = 0.415). At the protein level, VEGF was also upregulated by mechanical force application, independent of the O_2_ supply (*P* ≤ 0.032, Fig. 3d). Similar to VEGF gene expression, VEGF protein expression during pressure application showed no additional changes after O_2_ restriction (*P* = 0.330).

Finally, hypoxia-inducible factor 1α (HIF-1α) was significantly stabilized by mechanical compressive forces (mechanotransduction) during both normoxic (*P* = 0.045, Lumox®) and hypoxic conditions (*P* = 0.007, polystyrene) (Fig. 3e, f), whereas the level of the O_2_ supply had no significant additional stabilizing effect on HIF-1α (*P* = 0.416).

### Effects of mechanotransduction vs. hypoxia on RANKL/OPG expression in hPDL fibroblasts

**Fig. 4:**
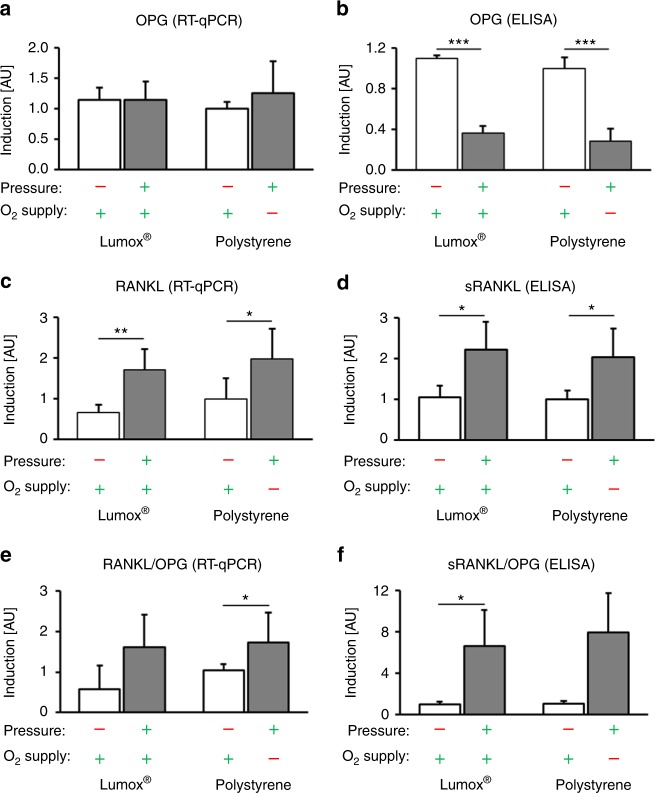
Effects of mechanotransduction vs. oxygen supply on hPDL fibroblast RANKL/OPG expression. **a** Gene and **b** protein expression of OPG in the presence of pressure under normoxic (Lumox®) and hypoxic (polystyrene) conditions (*N* = 3, *n* = 9). **c** Gene expression and the corresponding **d** protein secretion of (soluble) RANKL by hPDL fibroblasts (*N* = 2, *n* = 6). Calculated RANKL/OPG ratio for the **e** transcriptional and **f** protein levels. Bars indicate mean values ± standard deviation. *P ≤ 0.05, **P ≤ 0.01, ***P ≤ 0.001. AU = arbitrary units

The Lumox® and polystyrene control groups (in normoxia and no pressure) did not show significant RANKL or OPG expression differences (*P* ≥ 0.271). OPG gene expression in hPDL fibroblasts was not significantly affected by compressive mechanical strain, both in normoxic and hypoxic conditions (*P* ≥ 0.502, Fig. 4a). In contrast, OPG protein secretion from hPDL fibroblasts was significantly reduced during pressure application (mechanotransduction), and no effect of reduced O_2_ supply was observed (*P* ≤ 0.001, Fig. 4b). RANKL gene expression (Fig. 4c) and protein secretion (Fig. 4d) were enhanced by pressure application during normoxia (*P* ≤ 0.036, Lumox®) and hypoxia (polystyrene) (*P* ≤ 0.052). The level of the oxygen supply during compression, however, had no significant effect (*P* ≥ 0.813). The RANKL/OPG ratio at the transcriptional level showed a significant increase in the presence of mechanical compression and a restricted O_2_ supply (*P* = 0.013, Fig. 4e), whereas pressure application under normoxia (Lumox®) resulted in a tendency towards an increase in the RANKL/OPG ratio (*P* = 0.200). Altering the oxygen supply during pressure application did not show any effect on the RANKL/OPG mRNA ratio (*P* = 0.986). At the protein level, the sRANKL/OPG ratio showed a significant increase without oxygen restriction (*P* = 0.016) and a tendency towards increase during oxygen restriction (*P* = 0.052) (Fig. 4f). A change in the oxygen supply did not affect this observation (*P* = 0.928).

### Effects of mechanotransduction vs. hypoxia on membrane-bound RANKL protein expression and hPDL-mediated osteoclastogenesis

**Fig. 5:**
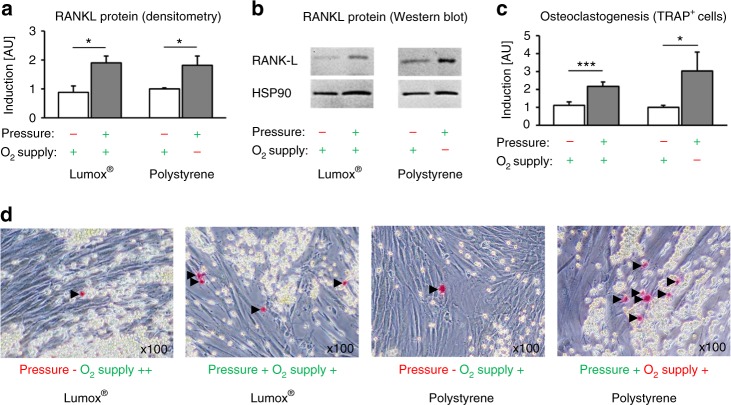
Effects of mechanotransduction vs. oxygen supply on hPDL-fibroblast-mediated osteoclastogenesis. **a** Densitometric immunoblot analysis of membrane-bound RANKL protein expression (*N* = 5). **b** Representative immunoblot of membrane-bound RANKL protein expression. **c** Quantification of TRAP-positive osteoclast-like cells per coculture well (*N* = 3, *n* = 9). **d** Representative images (×100) of coculture TRAP staining. TRAP-positive cells appear red (black arrows), RAW264.7 spherical osteoclast-precursor cells appear yellow and spindle-shaped hPDL fibroblasts are transparent. Bars indicate mean values ± standard deviation. *P ≤ 0.05, **P ≤ 0.01, ***P ≤ 0.001. AU = arbitrary units

The densitometric immunoblot (Western blot) analysis of membrane-bound RANKL protein expression showed a significant induction of protein expression by compressive force application (Fig. 5a, b) during normoxia (*P* = 0.001, Lumox®) and hypoxia (*P* = 0.014, polystyrene), which did not depend on the O_2_ supply (*P* = 0.964). This resulted in significantly increased hPDL-fibroblast-mediated osteoclastogenesis in the coculture after compressive force application (mechanotransduction), independently of the O_2_ supply (*P* ≤ 0.019, Fig. 5c, d). Hypoxic conditions during compression resulted in a slight additional increase in osteoclastogenesis, however, this was not significant (*P* = 0.298). The Lumox® and polystyrene control groups (in normoxia and no pressure) did not show significant differences in osteoclastogenesis (*P* = 0.775).

## Discussion

In this study, we investigated the relative importance of mechanotransduction and reduced O_2_ supply (hypoxia) to hPDL-fibroblast-mediated osteoclastogenesis during orthodontic tooth movement. The results from our in vitro experiments showed that the mechanical deformation of hPDL fibroblasts seemed to play a much more important role in the mediation of orthodontic tooth movement by hPDL fibroblasts at a cellular–molecular level than the concomitant reduction in the O_2_ supply.

The gene expression of COL1A2, which encodes the alpha-2 chain of collagen type I and is thus indicative of collagen synthesis, is very important for orthodontic tooth movement, considering that Type I collagen is the predominant collagen in the extracellular matrix of the periodontal ligament.^17^ COL1A2 was much more strongly expressed during mechanical loading, whereas a reduced O_2_ supply had no significant additional effect during loading. The same results were found for total collagen, which was also increased after the mechanical compression of hPDL fibroblasts independent of the oxygen supply. This indicates that the process of collagen synthesis might also be predominantly controlled by mechanotransduction. This finding is substantiated by the findings of Kook et al.^17^, who described the mechanical upregulation of Type I collagen by extracellular signal-regulated kinase and c-Jun N-terminal kinase, which transmitted mechanical signals into the nuclei of hPDL fibroblasts.

Orthodontic tooth movement involves the alteration of not only the collagen network but also the surrounding bone architecture. ALPL is highly expressed in and secreted by mature osteoblasts during bone formation; osteoblasts share ultrastructural and functional similarities with hPDL fibroblasts, and these can transform into osteoblasts.^18–20^ Osteoblast activity in the periodontal ligament is much higher than in other connective tissues.^21^ In our study, we observed increased ALPL expression at both the mRNA and protein levels after orthodontic compressive force application, which is similar to the findings of Nettelhoff et al.^22^ The restriction of the oxygen supply did not alter the force-induced expression of ALPL at all, indicating that ALPL is mainly mechanically regulated as well.

The primary response of hPDL fibroblasts to orthodontic forces is the synthesis and secretion of prostaglandins by COX-2, which in turn triggers osteoclastogenesis via the RANKL/OPG pathway.^1^ As already reported,^1,7,22^ orthodontic compressive force application led to enhanced COX-2 expression in our model of orthodontic tooth movement, whereas reduced oxygen levels did not alter this proinflammatory effect. PGE2, a product of COX-2,^23^ was also upregulated by the mechanical compression of hPDL fibroblasts independent of the O_2_ supply. This indicates the presence of a primarily mechanically triggered pathway causing inflammation in the context of orthodontic tooth movement.

VEGF is a growth factor involved in the neoformation and vasodilation of blood vessels.^24^ During orthodontic tooth movement, the formation of new blood vessels and the reshaping of existing blood vessels is induced in the periodontal ligament.^25^ Until now, it has been assumed that in the context of orthodontic tooth movement, VEGF is primarily upregulated by low oxygen levels due to a compression of blood vessels within the PDL.^13^ This mechanism is often explained by the dependence of VEGF expression on HIF-1α, as HIF-1α is stabilised by hypoxic conditions and VEGF is a target gene of HIF-1α.^26^ HIF-1α is of major importance for the adaptation of tissues to a reduced O_2_ supply.^27^ However, it has been proposed that HIF-1α can also be stabilised under normoxic conditions,^28^ as was the case in our study in the context of OTM, which would thus explain the mechanically induced upregulation of VEGF expression independent of the O_2_ supply. Our results confirm this assumption and indicate that the upregulation of VEGF is mainly mechanical. Li et al.^29^ described the enhancement of the expression of VEGF by either compression or hypoxia and the additive effect resulting from the combination of both stimuli; however, the upregulation of VEGF resulted only from compression according to Miyagawa.^25^ An inflammatory elevation of VEGF by lipopolysaccharides has been reported as well.^30^ Considering all these findings, one must assume that the expression of VEGF at both the mRNA and protein levels, and especially the processes of the neoformation and vasodilation of blood vessels, are not only regulated by oxygen levels but also predominantly by mechanical forces and the resulting pseudo-inflammatory processes during OTM.

The signalling molecule HIF-1α has often been used as a marker for cells under hypoxic conditions.^13,27^ Whereas the canonical method of HIF-1α stabilisation is via hypoxia,^31,32^ there have been reports that HIF-1α stabilisation is also possible by non-canonical methods,^32^ including Toll-like receptor activation by bacterial lipopolysaccharides,^30,33^ which occurs during periodontitis,^8,9,34^ and by mechanotransduction, which has been reported in endothelial cells.^31^ Our model of simulated orthodontic compressive forces showed the predominantly mechanical stabilisation of HIF-1α, indicating that in the context of orthodontic tooth movement, HIF-1α might be stabilised mechanically rather than by hypoxia. Considering that many of the investigated genes, such as COX-2 and VEGF, are target genes of HIF-1α,^9,30,35^ and that even the RANKL/OPG system and osteoclastogenesis are influenced by HIF-1α via the proinflammatory pathway consisting of COX-2, PGE2 and RANKL,^36^ HIF-1α seems to be a key factor involved in the complex regulation of orthodontic tooth movement. Feng et al.^31^ observed the mechanical stabilisation of HIF-1α in endothelial cells in blood vessels via the deubiquitinating enzyme Cezanne. This pathway could possibly be responsible for the mechanical stabilisation of HIF-1α in hPDL fibroblasts during orthodontic tooth movement, which merits further investigation.

The TNF-related ligand RANKL and its decoy receptor osteoprotegerin (OPG) both play important roles in the regulation of bone remodelling,^37^ orthodontic tooth movement and root resorption.^38^ RANKL is responsible for the differentiation of osteoclast-precursor cells and the activation of premature osteoclasts.^38^ The enhanced expression of both the soluble and membrane-bound subtypes of RANKL was observed after orthodontic compressive force application, indicating that increased osteoclastogenesis and bone resorption occurred in the compression areas of the periodontal ligament. A reduced O_2_ supply, on the other hand, did not alter the force-induced expression of RANKL of either subtype. In line with that finding, OPG secretion was only reduced by compressive force application and was not altered by a restricted oxygen supply. Whereas the regulation of RANKL and OPG is controlled by many factors, there is a connection between RANKL and HIF-1α via the proinflammatory pathway involving COX-2 and PG-E_2_,^36^ which upregulate RANKL. This might be one of several reasons why the RANKL/OPG pathway is mainly affected by mechanical loading rather than by reduced oxygen levels.

An increased RANKL/OPG ratio also resulted in more pronounced osteoclastogenesis under compressive forces. This effect, which has already been reported in the literature,^7,24,25^ was not affected by hypoxia, indicating that the signalling pathway resulting in osteoclastogenesis is mainly regulated by mechanical compression. Li et al.^29^ however observed the significant enhancement of osteoclast formation by compression as well as by hypoxia. The number of osteoclast-like cells was significantly increased after 6 h of compression than after 6 h of hypoxia, which is in line with our results that show that mechanical loading is the main driving force for osteoclastogenesis. The discrepancy between the compression group and the hypoxia group in their study, however, diminished after 24 and 72 h of stimulation, which suggests that hypoxia may induce hPDL-fibroblast-mediated osteoclastogenesis in a slower but more lasting manner compared with that induced by mechanical loading. The effect of either compression or hypoxia may thus be time-dependent, with hypoxic effects manifesting themselves at a later time. However, in our experiments, we did not evaluate the effects of hypoxia by itself, as did Li et al.^29^, but rather the additional effects of hypoxia in combination with mechanical loading compared with the effects of mechanical loading only, for the first time. Li et al.^29^ also used much higher compressive forces of 25 g/cm^2^ and much more pronounced hypoxia, with a residual oxygen concentration of only 2%, compared with those used in our experiments, which limits the comparability of the results. We also used murine RAW cells instead of human peripheral blood cells in the cocultures with human fibroblasts, which is an established and reliable model for studying fibroblast-mediated osteoclastogenesis, as shown before by our own^6,15,16,39,40^ and other studies.^41,42^

Both cell number and viability were reduced by compressive forces in our experiments. This effect, also reported in other literature,^22,43^ was enhanced even under hypoxic conditions and indicated that both mechanotransduction and hypoxia could reduce the number and viability of hPDL fibroblasts, which, in case of mechanotransduction, is most likely an artificial effect of the in vitro model, as previously reported.^16^

## Conclusions

- The cellular and molecular mediation of osteoclastogenesis during orthodontic tooth movement by hPDL fibroblasts seems to be mainly regulated by the application of force (mechanotransduction), whereas hypoxic effects appear to play only a minor role, since they had no significant additional effects on the expression patterns of hPDL fibroblasts and the associated osteoclastogenesis.
- The hypoxic marker HIF-1α, which has many target genes such as COX-2 and VEGF, seems to be stabilised mainly by mechanical loading rather than hypoxia in the context of orthodontic tooth movement.

## Materials and methods

### In vitro cell culture experiments

**Fig. 6:**
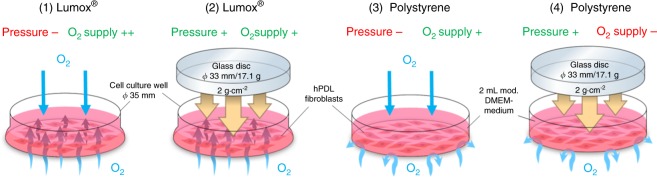
Set-up used for the hPDL fibroblast experiments to evaluate the four experimental groups (1–4)

In vitro cell culture experiments and methods were performed and reported as previously published.^6,15,16,39,44^ hPDL fibroblasts were obtained from the periodontal connective tissue of human teeth that were free of decay and were extracted for medical reasons. All experiments were performed in accordance with the relevant guidelines and regulations. Approval for the collection and usage of hPDL fibroblasts was obtained from the ethics committee of the University of Regensburg, Germany (approval number 12-170-0150). Briefly, we cultivated the tissue samples in six-well cell culture plates (37 °C, 5% CO_2_, 100% H_2_O) in complete media (high-glucose DMEM, D5796, Sigma-Aldrich®, St. Louis, MI, USA) with 10% FCS (P30-3306, PAN-Biotech, Aidenbach, Germany), 1% L-glutamine (SH30034.01, GE Healthcare Europe, Munich, Germany), 100 µmol·L^−1^ ascorbic acid (A8960, Sigma-Aldrich^®^) and 1% antibiotics/antimycotics (A5955, Sigma-Aldrich^®^) until proliferatory outgrowth of adherently growing fibroblasts was observed. The cells were characterised by hPDL-specific marker genes and a spindle-shaped morphology, as reported previously.^15,44^ For the in vitro study, hPDL fibroblasts from the third to fifth passages that were pooled from six individuals (three male, three female, ages 17–27 years) were seeded at a density of 2000 cells per mm^2^ into either standard six-well cell culture plates without oxygen permeability in the polystyrene base/membrane (353046, BD, Heidelberg, Germany) or in special gas-permeable Lumox® dishes (94.6077.331, Sarstedt, Nürnbrecht, Germany) with ultra-thin gas-permeable bases/membranes, which provided a continuous oxygen supply to the adherently growing fibroblasts at the base (Fig. 6).

### Experimental set-up

To simulate mechanical orthodontic strain in hPDL fibroblasts in compression areas of the periodontal ligament, a physiological orthodontic pressure of 2 g·cm^–2^ was applied under specific cell culture conditions (37 °C, 5% CO_2_, 100% water-saturated, 2 ml DMEM/well) for 48 h after a preincubation phase of 24 h by means of a sterilised glass disc according to an established and published method.^6,7,15,16,39,44^ (Fig. 6) The following four experimental groups with 6–9 biological replicates (samples) each (*n*) during 2–3 consecutive experiments (*N*) with three replicates each were incubated at 70% confluency for a total of 72 h: (1) no mechanical orthodontic compressive strain + normoxia (control, Lumox®); (2) mechanical strain + normoxia (Lumox®); (3) no mechanical strain + normoxia (control, polystyrene); (4) mechanical strain + hypoxia (polystyrene). In the conventional, non-gas-permeable polystyrene cell culture plates and in vivo, the applied compressive forces not only induced mechanical deformation and stress in the adherently growing hPDL fibroblasts but also limited the oxygen supply (group 4), which was not the case in the Lumox® plates with an intact oxygen supply via the gas-permeable membrane (group 2) (Fig. 6). This experimental set-up allowed for the experimental isolation and separation of the mechanotransducive and hypoxic effects that occur concomitantly during OTM, thus enabling an investigation of their respective importance.

### Determination of cell number and cell viability via MTT assays

The cell number per cm^2^ was determined after 72 h of incubation with a Beckman Coulter Counter Z2™ (Beckman Coulter GmbH, Krefeld, Germany). The cell viability of the hPDL fibroblasts was determined for all experimental groups by MTT (3-(4,5-dimethylthiazol-2-yl)-2,5-diphenyltetrazolium bromide) assays. For the final 5 h of the 72 h incubation phase, 400 µL of MTT solution in PBS (5 mg•mL^–1^, 4022.1, Carl Roth GmbH & Co. KG, Karlsruhe, Germany) was added per well. After the removal of the medium, 1 mL of DMSO per well was added. The hPDL fibroblasts were then incubated for another 5 min at 37 °C, and the absorbance was quantified at 550 nm by means of an ELISA reader (Multiscan GO Microplate Spectrophotometer, Thermo Fisher Scientific Inc., Schwerte, Germany), which corresponded to the cell viability.

### Determination of the relative gene expression via quantitative real-time polymerase chain reaction

We quantified the expression of genes involved in inflammation (COX-2), collagen synthesis (COL1A2), angiogenesis (VEGF) and osteoblastogenesis (ALPL), and the expression of RANKL and its decoy receptor osteoprotegerin (OPG), which are the most important signalling molecules in the process of osteoclastogenesis.^37^

The RNA isolation and quality assessment was performed as described previously according to MIQE guidelines.^44,45^ Briefly, we isolated total RNA from hPDL fibroblasts by adding 1 mL of peqGOLD TriFast^TM^ (PEQLAB Biotechnology GmbH, Erlangen, Germany) per well and performing the isolation according to the manufacturer’s instructions. The RNA pellet was eluted in 25 µL of nuclease-free water (T143, Bioscience-Grade, Carl Roth GmbH & Co. KG). The utilised extraction protocol ensured good RNA integrity (RIN, 28 S/18 S ratio) and the absence of genomic DNA and contamination, as shown previously.^44^ For the purity assessment and the quantification of the eluted total RNA, the optical density (OD) was photometrically measured at 280 , 260 and 230 nm (NanoDrop ND-2000, Thermo Fisher Scientific Inc.), with an OD_260nm_ value of 1 representing 40 ng/µL total RNA. An OD_260 nm/280 nm_ ratio of >1.8 indicated protein-free RNA, and an OD_260 nm/230 nm_ ratio of >2.0 indicated phenol-/ethanol-free RNA.^44^

For cDNA synthesis, we used a standard amount of 1 μg of RNA per sample and transcribed it into cDNA (incubation for 60 min at 37 °C) by using 0.1 nmol of an oligo-dT18 primer (1 µL, SO131, Life Technologies, Thermo Fisher Scientific Inc.), 0.1 nmol of a random hexamer primer (1 µL, SO142, Life Technologies), 40 nmol dNTP mix (1 µL, 10 nmol per dNTP, Roti®-Mix PCR3, L785.2), 4 µL of 5× M-MLV-buffer (M1705, Promega, Fitchburg, WI, USA), 40 U (1 µL) of an RNase inhibitor (EO0381, Life Technologies) and 200 U (1 µL) reverse transcriptase (M1705, Promega) in 20 µL of nuclease-free H_2_O (Roth BioScience Grade T143, Carl Roth GmbH & Co. KG). After the heat inactivation of the reverse transcriptase (95 °C, 2 min), the first-strand cDNA was diluted 1:5 and stored until use at −20 °C. To minimise experimental variation, cDNA synthesis was performed for all samples at the same time.

For RT-qPCR amplification, we used the Mastercycler® ep Realplex-S thermocycler (Eppendorf AG, Hamburg, Germany). For each reaction, we mixed 7.5 µL of SYBR® Green JumpStart™ Taq ReadyMix™ (S4438, Sigma Aldrich®), 7.5 pmol (0.75 µL) of the respective primer pair (3.75 pmol per primer) and 1.5 µL of the respective diluted cDNA, and then added nuclease-free H_2_O (BioScience Grade T143, Carl Roth GmbH & Co. KG) to bring the total volume to 15 µL. To avoid technical errors during manual pipetting, all components except the cDNA solution were prepared as a master mix. cDNA amplification was performed with 45 cycles (initial heat activation 95 °C/5 min, per cycle 95 °C/10 s of denaturation, 60 °C/8 s of annealing and 72 °C/8 s of extension). The SYBR Green I fluorescence was quantified at 521 nm at the end of each extension step. The *C*_q_ values were determined as the second derivative maximum of the fluorescence signal curve with the software Realplex (version 2.2, Eppendorf AG, CalqPlex algorithm, Automatic Baseline, Drift Correction On). For the normalisation of the target genes (relative gene expression), we used a set of two reference genes (RPL22 and PPIB), which have been shown to be stably expressed in hPDL fibroblasts under the conditions investigated.^44^ The relative gene expression used for the statistical analysis was calculated as 2^–ΔCq^, with ∆*C*_q_ = *C*_q_ (target gene) – *C*_q_ (mean RPL22/PPIB), divided by the respective 2^–Δ*C*q^ arithmetic mean of the Lumox® normoxic control group to set the relative gene expression to 1.^15,16^

We designed all primers (Table 1) according to the MIQE quality guidelines^45^ and previously described criteria^44^ by using NCBI PrimerBLAST and additional software to avoid the formation of dimers and secondary structures at the annealing temperature. The unmodified primers were synthesised and purified by Eurofins MWG Operon LLC (Huntsville, AL, USA; High Purity Salt Free Purification HPSF®). We performed a no-template control (NTC) without cDNA to assess possible faults resulting from primer dimers or contaminating DNA. The primer specificity was validated as described previously (melting-curve analysis and agarose gel electrophoresis).^44^

**Table 1:** RT-qPCR gene, primer, target and amplicon specifications for reference genes (PPIB, RPL22) and target genes

Gene symbol	Gene name (Homo sapiens)	Accession number^a^ (NCBI GenBank)	Chromosomal location^a^ (length)	5′-forward primer-3′ (length/ *T*_m_/ %GC/max. ∆*G* Hairpin &Self-Dimer/ Self-Comp./ Self-3′-Comp.)	5′-reverse primer-3′ (length/*T*_m_ /%GC/max. ∆*G* Hairpin &Self-Dimer/Self-Comp./ Self-3′-Comp.)	Primer location^b^ (max. ∆G Cross-Dimer)	Amplicon (length, %GC, *T*_m_, SSAT)	Amplicon location (bp of Start/Stop)	Intron spanning^b^ (length)	In silico qPCR specificity^c^	Variants targeted (transcript/splice)^b^
PPIB	Peptidylprolyl isomerase B	NM_000942.4	15q22.31 (1 045 bp)	TTCCATCGTGTAATCAAGGACTTC (24 bp/ 61.3 °C/ 41.7%/–1.3/4/ 2)	GCTCACCGTAGATGCTCTTTC (21 bp/ 61.2 °C/ 52.4%/–0.7/4 /0)	Exon ¾ (–2.1)	88 bp, 53.4%, 86.1 °C, no SSAT	446/533	Yes (3 194 bp)	Yes (BLAST/UCSC)	Yes
RPL22	Ribosomal protein L22	NM_000983.3	1p36.31 (2 099 bp)	TGATTGCACCCACCCTGTAG (20 bp/ 62.2 °C/ 55.0%/–3.4/4/2)	GGTTCCCAGCTTTTCCGTTC (20 bp/61.8 °C/55.0%/–3.0/ 4/ 0)	Exon 2/3 (–1.5)	98 bp, 44.9%, 83.8 °C, no SSAT	115/212	Yes (4 597 bp)	Yes (BLAST/UCSC)	Yes
ALPL	Alkaline phosphatase, liver/bone/kidney	NM_000478.5	1p36.12 (2 613 bp)	ACAAGCACTCCCACTTCATCTG (22 bp/ 60.3 °C/ 50.0%/–0.5/3/ 2)	GGTCCGTCACGTTGTTCCTG (20 bp/61.4 °C/60.0%/–3.3/ 5/ 1)	Exon 7–8/9 (–2.1)	132 bp, 56.1%, 89.5 °C, no SSAT	1 045/1 176	Yes (3 290 bp)	Yes (BLAST/UCSC)	Yes
COL1A2	Collagen type I alpha-2 chain	NM_000089.3	7q21.3 (5 411 bp)	AGAAACACGTCTGGCTAGGAG (21 bp/ 59.8 °C/52.4%/–3.3/4/2)	GCATGAAGGCAAGTTGGGTAG (21 bp/59.8 °C/52.4%/–2.3/5 /0)	Exon 50/51 (–0.7)	105 bp, 44.8%, 83.3 °C no SSAT	4 139/4 243	Yes (7 10 bp)	Yes (BLAST/UCSC)	Yes
COX-2 (PTGS2)	Prostaglandin-endoperoxide synthase 2	NM_000963.3	1q31.1 (4 507 bp)	GAGCAGGCAGATGAAATACCAGTC (24 bp/62.7 °C/ 50.0%/ 0.0/2/ 2)	TGTCACCATAGAGTGCTTCCAAC (23 bp/60.6 °C/47.8%/–1.3/4 /0)	Exon 8/9 (–3.2)	131 bp, 42.0%, 82.9 °C, no SSAT	1 457/1 587	Yes (4 86 bp)	Yes (BLAST/UCSC)	Yes
VEGFA	Vascular endothelial growth factor A	NM_001171623.1	6p21.1 (3 677 bp)	TGCAGACCAAAGAAAGATAGAGC (23 bp/ 58.9 °C/ 43.5%/–3.4/4/ 2)	ACGCTCCAGGACTTATACCG (20 bp/59.4 °C/55.0%/–1.3/ 5/ 2)	Exon 5–6/7 (–3.3)	107 bp, 43.9%, 83.7 °C, no SSAT	1 426/1 532	No	Yes (BLAST/UCSC)	Yes
TNFRSF11B (OPG)	Tumour necrosis factor receptor superfamily member 11b (osteoprotegerin)	NM_002546.3	8q24.12 (2 354 bp)	TGTCTTTGGTCTCCTGCTAACTC (23 bp/ 60.6 °C/47.8%/0.0/2/ 0)	CCTGAAGAATGCCTCCTCACAC (22 bp/62.1 °C/54.6%/–0.9/4 /0)	Exon 3/4 (–1.8)	124 bp, 42.7%, 83.1 °C no SSAT	824/947	Yes (2 019 bp)	Yes (BLAST/UCSC)	Yes
TNFSF11 (RANKL)	Tumour necrosis factor superfamily member 11 (receptor activator of nuclear factor kappa B)	NM_003701.3	13q14.11 (2 216 bp)	ATACCCTGATGAAAGGAGGA (20 bp/54.9 °C/45.0%/–1.3/3/ 0)	GGGGCTCAATCTATATCTCG (20 bp/54.6 °C/50.0%/–0.5/ 4/ 2)	Exon 8 (–2.0)	202 bp, 43.6%, 82.5 °C, no SSAT	907/1 108	No	Yes (BLAST/UCSC)	Yes

Primer secondary structures (hairpin, dimers) at annealing temperature were determined by BeaconDesigner (Premier Biosoft International, http://www.premierbiosoft.com/qOligo/Oligo.jsp?PID = 1). Primers for TNFSF11 were previously published by Nettelhoff et al.^22^

*T*_*m*_, melting temperature of primer/specific qPCR product (amplicon); *%GC,* guanine/cytosine content; *bp,* base pairs; *Comp.,* complementarity; *SSAT,* secondary structure at annealing temperature (determined with UNAFold; https://eu.idtdna.com/UNAFold?)

^a^Primer design based on this sequence. The database source was the NCBI Nucleotide database (http://www.ncbi.nlm.nih.gov/nuccore)

^b^Determined with PrimerCheck (http://projects.insilico.us/SpliceCenter/PrimerCheck; SpliceCenter)

^c^Determined in silico by NCBI PrimerBLAST (https://www.ncbi.nlm.nih.gov/tools/primer-blast) and UCSC In-Silico PCR (http://genome.ucsc.edu/cgi-bin/hgPcr)

### Enzyme-linked immunosorbent assays

For the quantification of OPG, soluble RANKL, ALPL, prostaglandin E2 (PGE2) and VEGF protein secretion in the hPDL cell supernatant, we used commercially available ELISA kits according to the manufacturers’ instructions (OPG: EHTNFRSF11B, Thermo Fisher Scientific Inc.; sRANKL: RD193004200R; Biovendor, Brno, Czech Republic; ALPL: OKEH00757; Aviva Systems, San Diego, USA; PGE2: 514010; Cayman Chemicals, Ann Arbor, USA; VEGF-A: RAB0507, Sigma Aldrich). We used cell culture supernatants from two independent experiments (*N* = 2) with a total of six biological replicates (*n* = 6). For the ELISA of OPG, we diluted the cell supernatants 1:10 in appropriate dilution buffer. The protein expression per well was related to the respective number of hPDL fibroblasts, as counted with a Beckman Coulter Counter Z2™ (Beckman Coulter GmbH).

### Quantification of total collagen in the cell culture supernatant

For the quantification of total collagen, we used a commercially available kit (K218-100, Biovision, Milpitas, USA) according to the manufacturer’s instructions.

### Quantification of RANKL and HIF-1α stabilization via western blot

Since RANKL can be expressed as two subtypes—soluble and membrane-bound—we also investigated the expression of membrane-bound RANKL by performing immunoblotting with a RANKL-specific antibody. In addition, we assessed the stability of HIF-1α, which, among other target genes, regulates COX-2 and VEGF expression.^26,35^ Total protein from hPDL fibroblasts was isolated with 100 µL of CelLytic™ M per well (C2978; Sigma-Aldrich®) supplemented with proteinase inhibitors (Carl Roth GmbH & Co. KG). To reduce proteinase activity, the proteins were kept on ice for the entire procedure. The determination of protein concentration was performed with RotiQuant (K015.3; Carl Roth GmbH & Co. KG) according to the manufacturer’s instructions. For immunoblotting, we separated equal amounts of total protein on a 10% SDS-polyacrylamide (RANKL) or 8% SDS-polyacrylamide (HIF-1α) gel under reducing conditions and transferred the proteins onto polyvinylidene difluoride (PVDF) membranes via electroblotting. To reduce the nonspecific binding of antibodies, we blocked the membranes with 5% nonfat milk in Tris-buffered saline and 0.1% Tween 20, pH 7.5 (TBS-T), at 4 °C overnight. Then, we incubated the membranes with anti-RANKL (1:2 000, ABIN500805, Antibodies-Online, Aachen, Germany), anti-HIF-1α (1:2 000, Santa Cruz Biotech, Heidelberg, Germany), anti-HSP90 (reference, 1:500, Santa Cruz Biotech) and anti-β-actin (reference, 1:5 000, Sigma-Aldrich®) for 1 h at room temperature. After washing three times in TBS-T, we incubated the blots for another 1 h with horseradish peroxidase-conjugated anti-rabbit IgG (Pierce, Rockford, USA) diluted 1:5 000 in 0.5% milk in TBS-T at room temperature. We visualised the antibody binding by using an enhanced chemiluminescence system (Pierce, Rockford, USA).

### TRAP histochemistry (hPDL-mediated osteoclastogenesis)

To investigate the effect of mechanotransduction vs. that of hypoxia on the mediation of osteoclastogenesis by hPDL fibroblasts during orthodontic tooth movement, we performed coculture experiments with osteoclast-precursor cells. At the end of the total 72-h incubation period, hPDL fibroblasts from each experimental group were washed (PBS), and a macrophage osteoclast-precursor cell line (immortal murine RAW264.7 cells, CLS Cell Lines Service, Eppelheim, Germany) was added after force application at a concentration of 70 000 cells per well, thus avoiding the potential force-induced induction of RAW cell differentiation that was not mediated by RANKL.^39^ The resulting coculture was then incubated for another 72 h under specific cell culture conditions.^6,39^ Histochemical TRAP staining (red) was used to detect differentiated osteoclast-like cells.^46^ TRAP-positive cells were quantified at a magnification of ×100 with an Olympus IX50 microscope (Olympus, Germany) in ten random fields of view per well (biological replicates) by a blinded observer, and the arithmetic mean was used for further analysis.

### Statistical analysis

Prior to the statistical analysis, all absolute data values were divided by the respective arithmetic mean of the Lumox® normoxic control group to obtain normalised data values relative to the values of the controls, which were set to 1. Using the software application SPSS® Statistics 24 (IBM^®^, Armonk, NY, USA), all data were tested for a normal distribution (Shapiro–Wilk test, visual assessment of histograms) and the homogeneity of variance (Levene’s test). The descriptive statistics are given as the mean (M) ± standard deviation (SD). The normal distribution of all data was confirmed. The experimental groups were compared by one-way ANOVA and validated by Welch’s test, since the homogeneity of variance was not always present. We used Games–Howell post hoc tests for heterogeneous variances in pairwise comparisons. Statistical significance was assumed at *P* ≤ 0.05.

## References

[1] Meikle MC (2006). The tissue, cellular, and molecular regulation of orthodontic tooth movement: 100 years after Carl Sandstedt. Eur. J. Orthod..

[2] Lekic P, McCulloch CA (1996). Periodontal ligament cell population: the central role of fibroblasts in creating a unique tissue. Anat. Rec..

[3] He Y, Macarak EJ, Korostoff JM, Howard PS (2004). Compression and tension: differential effects on matrix accumulation by periodontal ligament fibroblasts in vitro. Connect. Tissue Res..

[4] Wolf M (2016). CD8+ T cells mediate the regenerative PTH effect in hPDL cells via Wnt10b signaling. Innate Immun..

[5] Kirschneck C, Maurer M, Wolf M, Reicheneder C, Proff P (2017). Regular nicotine intake increased tooth movement velocity, osteoclastogenesis and orthodontically induced dental root resorptions in a rat model. Int. J. Oral. Sci..

[6] Kirschneck C, Proff P, Maurer M, Reicheneder C, Romer P (2015). Orthodontic forces add to nicotine-induced loss of periodontal bone: an *in vivo* and *in vitro* study. J. Orofac. Orthop..

[7] Kanzaki H, Chiba M, Shimizu Y, Mitani H (2002). Periodontal ligament cells under mechanical stress induce osteoclastogenesis by receptor activator of nuclear factor kappaB ligand up-regulation via prostaglandin E2 synthesis. J. Bone Miner. Res..

[8] Golz L (2014). LPS from *P. gingivalis* and hypoxia increases oxidative stress in periodontal ligament fibroblasts and contributes to periodontitis. Mediators Inflamm..

[9] Golz L (2015). Hypoxia and *P. gingivalis* synergistically induce HIF-1 and NF-kappaB activation in PDL cells and periodontal diseases. Mediators Inflamm..

[10] Son GY, Shin DM, Hong JH (2015). Bacterial PAMPs and allergens trigger increase in [Ca(2+)]i-induced cytokine expression in human PDL fibroblasts. Korean J. Physiol. Pharmacol..

[11] Basdra EK (1997). Biological reactions to orthodontic tooth movement. J. Orofac. Orthop..

[12] Khouw FE, Goldhaber P (1970). Changes in vasculature of the periodontium associated with tooth movement in the rhesus monkey and dog. Arch. Oral. Biol..

[13] Niklas A, Proff P, Gosau M, Romer P (2013). The role of hypoxia in orthodontic tooth movement. Int. J. Dent..

[14] Kanzaki H (2006). Cyclical tensile force on periodontal ligament cells inhibits osteoclastogenesis through OPG induction. J. Dent. Res..

[15] Schroder A (2018). Expression kinetics of human periodontal ligament fibroblasts in the early phases of orthodontic tooth movement. J. Orofac. Orthop..

[16] Schröder A (2018). Effects of ethanol on human periodontal ligament fibroblasts subjected to static compressive force. Alcohol.

[17] Kook SH (2009). Mechanical force induces type I collagen expression in human periodontal ligament fibroblasts through activation of ERK/JNK and AP-1. J. Cell. Biochem..

[18] Lallier TE, Spencer A, Fowler MM (2005). Transcript profiling of periodontal fibroblasts and osteoblasts. J. Periodontol..

[19] Jiang N (2016). Periodontal ligament and alveolar bone in health and adaptation: tooth movement. Front. Oral. Biol..

[20] Alberts, B. et al. *Fibroblasts and their Transformations: the Connective-Tissue Cell Family*. 4th edn (Garland Science, New York, 2002).

[21] Yamaguchi M, Shimizu N, Shibata Y, Abiko Y (1996). Effects of different magnitudes of tension-force on alkaline phosphatase activity in periodontal ligament cells. J. Dent. Res..

[22] Nettelhoff L (2016). Influence of mechanical compression on human periodontal ligament fibroblasts and osteoblasts. Clin. Oral. Investig..

[23] Shimizu N (1998). Induction of COX-2 expression by mechanical tension force in human periodontal ligament cells. J. Periodontol..

[24] Di Domenico M (2012). Cytokines and VEGF induction in orthodontic movement in animal models. J. Biomed. Biotechnol..

[25] Miyagawa A, Chiba M, Hayashi H, Igarashi K (2009). Compressive force induces VEGF production in periodontal tissues. J. Dent. Res..

[26] Liu W, Shen SM, Zhao XY, Chen GQ (2012). Targeted genes and interacting proteins of hypoxia inducible factor-1. Int. J. Biochem. Mol. Biol..

[27] Ke Q, Costa M (2006). Hypoxia-inducible factor-1 (HIF-1). Mol. Pharmacol..

[28] Kuschel A, Simon P, Tug S (2012). Functional regulation of HIF-1alpha under normoxia-is there more than post-translational regulation?. J. Cell. Physiol..

[29] Li ML (2016). Compression and hypoxia play independent roles while having combinative effects in the osteoclastogenesis induced by periodontal ligament cells. Angle Orthod..

[30] Li JP (2012). Lipopolysaccharide and hypoxia-induced HIF-1 activation in human gingival fibroblasts. J. Periodontol..

[31] Feng S (2017). Mechanical activation of hypoxia-inducible factor 1alpha drives endothelial dysfunction at atheroprone sites. Arterioscler. Thromb. Vasc. Biol..

[32] Masoud GN, Li W (2015). HIF-1α pathway: role, regulation and intervention for cancer therapy. Acta Pharm. Sin. B.

[33] Jantsch J (2011). Toll-like receptor activation and hypoxia use distinct signaling pathways to stabilize hypoxia-inducible factor 1alpha (HIF1A) and result in differential HIF1A-dependent gene expression. J. Leukoc. Biol..

[34] Frede S, Stockmann C, Freitag P, Fandrey J (2006). Bacterial lipopolysaccharide induces HIF-1 activation in human monocytes via p44/42 MAPK and NF-kappaB. Biochem. J..

[35] Kaidi A, Qualtrough D, Williams AC, Paraskeva C (2006). Direct transcriptional up-regulation of cyclooxygenase-2 by hypoxia-inducible factor (HIF)-1 promotes colorectal tumor cell survival and enhances HIF-1 transcriptional activity during hypoxia. Cancer Res..

[36] Nogueira AV (2014). Biomechanical loading modulates proinflammatory and bone resorptive mediators in bacterial-stimulated PDL cells. Mediators Inflamm..

[37] Proff P, Romer P (2009). The molecular mechanism behind bone remodelling: a review. Clin. Oral Investig..

[38] Tyrovola JB, Spyropoulos MN, Makou M, Perrea D (2008). Root resorption and the OPG/RANKL/RANK system: a mini review. J. Oral. Sci..

[39] Kirschneck C, Meier M, Bauer K, Proff P, Fanghanel J (2017). Meloxicam medication reduces orthodontically induced dental root resorption and tooth movement velocity: a combined in vivo and in vitro study of dental-periodontal cells and tissue. Cell Tissue Res..

[40] Kirschneck C (2019). Effects of the highly COX-2-selective analgesic NSAID etoricoxib on human periodontal ligament fibroblasts during compressive orthodontic mechanical strain. Mediators Inflamm..

[41] Voisin V, Caballé-Serrano J, Sculean A, Gruber R (2016). Palatal fibroblasts reduce osteoclastogenesis in murine bone marrow cultures. BMC Oral. Health.

[42] Hase H, Kanno Y, Kojima H, Sakurai D, Kobata T (2008). Coculture of osteoclast precursors with rheumatoid synovial fibroblasts induces osteoclastogenesis via transforming growth factor beta-mediated down-regulation of osteoprotegerin. Arthritis Rheum..

[43] Kanjanamekanant K, Luckprom P, Pavasant P (2013). Mechanical stress-induced interleukin-1beta expression through adenosine triphosphate/P2X7 receptor activation in human periodontal ligament cells. J. Periodontal Res..

[44] Kirschneck C (2017). Valid gene expression normalization by RT-qPCR in studies on hPDL fibroblasts with focus on orthodontic tooth movement and periodontitis. Sci. Rep..

[45] Bustin SA (2009). The MIQE guidelines: minimum information for publication of quantitative real-time PCR experiments. Clin. Chem..

[46] Collin-Osdoby P, Yu X, Zheng H, Osdoby P (2003). RANKL-mediated osteoclast formation from murine RAW 264.7 cells. Methods Mol. Med..

